# The Present Shock and Time Re-appropriation in the Pandemic Era: Missed Opportunities for Science Education

**DOI:** 10.1007/s11191-020-00159-x

**Published:** 2020-09-25

**Authors:** Olivia Levrini, Paola Fantini, Eleonora Barelli, Laura Branchetti, Sara Satanassi, Giulia Tasquier

**Affiliations:** 1 Department of Physics and Astronomy, Alma Mater Studiorum – University of Bologna, Via Irnerio 46, 40126 Bologna, Italy; 2 Department of Mathematical, Physical and Computer Sciences, University of Parma, Parma, Italy

## Abstract

The crisis due to the COVID-19 pandemic led most people all over the world to deal with a change in their perception and organization of time. This happened also, and mainly, within the educational institutions, where students and teachers had to rearrange their teaching/learning dynamics because of the forced education at a distance. In this paper, we present an exploratory qualitative study with secondary school students aimed to investigate how they were experiencing their learning during lockdown and how, in particular, learning of science contributed to rearranging their daily lifetime rituals. In order to design and carry out our investigation, we borrowed constructs coming from a research field rather unusual for science education: the field of *sociology of time*. The main result concerns the discovery of the potential of the dichotomy between *alienation from time* and *time re-appropriation.* The former is a construct elaborated by the sociologist Hartmut Rosa to describe the society of acceleration in the “era of *future shock*”. The latter represents an elaboration of the construct of *appropriation* that the authors had operationally defined, starting from Bakhtin’s original idea, to describe the nexus between physics learning and identity*.* Thanks to the elaboration of the notion of *time re-appropriation* as feature of the “era of *present shock*”, the study unveils how school science, instead of preparing the young to navigate our fast-changing and complex society, tends to create “bubbles of rituals” that detach learning from societal concern.

## Introduction

We have been experiencing an extraordinary period, during which many parts of the world have been locked down due to the coronavirus infection. This paper aims to respond to the challenge launched by *Science & Education* journal (Erduran 2020) and tries to offer a perspective to critically reflect on what lessons can we learn from the pandemic and how can we rethink, accordingly, on science teaching and its role to form the young generation in this fast-changing, global, and complex world. Reiss, in his recent paper on this journal, discusses crucial topics that regard the history, philosophy, and sociology of science that can contribute to science education in the era of the COVID-19 pandemic (Reiss 2020). In our paper, we address the theme from a deeply different perspective: we will not focus on the scientific contents that should inform the curricula, but we will focus on “time rituals” in science learning that characterize schools and science classes and that the pandemic and lockdown have unveiled and challenged.

The study was inspired by the rather trivial observation that pandemic and lockdown let most of the people to and experience a significant change in their perception and organization of daily and social time. This happened also, and mainly, in schools, where students and teachers had to rearrange very quickly their ways to interact and, specifically, the space and time structures that characterize the learning and teaching dynamics (da Silva Vieira and Barbosa 2020). We retained that this type of change was interesting to analyse for at least two reasons: when things are rapidly changing, implicit dynamics and structures emerge more easily and, secondly, such changes can lead to new school scenarios that are worth to be anticipated. We then decided to carry out an exploratory qualitative study with secondary school students aimed to investigate how they were experiencing time during lockdown and if and how the pandemic crisis was changing their rituals in learning. Because of our specific research background, we focused on their learning in physics and mathematics and mainly investigated how time perceptions and eventual changes in learning rituals were related to the study of physics and mathematics. In the design and implementation of our investigation, we borrowed constructs coming from the field of *sociology of time*.

This field assumes that time, as well as being a scientific, anthropological, and psychological construct, can also be a sociological perspective, investing social forms and structures (Cavalli 1985). In fact, social actions and interactions, cultures, institutional and power dynamics can be analysed for their temporal structures, like rituals, images of the futures and relations with the past (Rosa 2010). Furthermore, social transformations can be understood by zooming in on individuals and how they perceive, represent, experience, and re-elaborate time (Leccardi 2009). Time perspective, from a sociological perspective, is a multiform lens that keeps together different scales, from the individual microcosmos to the social macrocosmos that retroact on each other (Rosa 2010). Very often these time structures are invisible, taken for granted and made trivial by habits and routines. Only in some historical moments do they emerge: one of these occasions is the pandemic era; when global time is accelerated by a natural phenomenon (the virus evolution), social institutions (policy, health systems, educational systems, economics) try to run as quickly as possible to slow down the epidemic velocity, and individual time is suspended in a bubble of present (a period of waiting).

During this period, the dynamics—and inner velocities—of natural phenomena, social practices, biological and cultural needs, daily rituals, and intersubjective and affective relationships are unveiled, and we tend to feel a deep sense of disruption due to their emergence. We will call this feeling “present shock,” and in this paper, we will try to describe it in comparison with the much better-known psychological state defined by sociologists as “future shock”. More relevantly for our research field, we will try to show both the inspirational and operational potential of these notions to capture learning dynamics and rituals in science education.

The paper is articulated as follows. In Section 2 our research framework is presented. Specifically, in Sections 2.1 we introduce the concept of *future shock* (Toffler and Toffler 1970) and its elaboration in sociology. Its presentation is followed by the description of the state of art in science education on research themes related to the development of future thinking and foresight skills. In section 2.2 we articulate the notion of *present shock* in analogy with the notion of future shock and formulate the research hypothesis that guided the empirical study. Special emphasis is given to the construct of *appropriation* elaborated in science education that has been used as theoretical lens to interpret the collected data. Sections 3 and 4 contain the qualitative study with secondary school students, which we carried out to investigate how they were experiencing time during the lockdown period. The results will contribute to unveiling how school science tends to create “bubbles of rituals” that detach learning from societal concerns. These results will allow us also to point out how, on the contrary, science education could equip secondary school students with a compass to navigate the complexity and uncertainty of our fast-changing society.

## Framework

### “Future Shock” and Alienation from Time

In 1965, the writer and futurist Alvin Toffler coined the term “future shock” to indicate a specific psychological state. Basing on the popular notion of “culture shock”, as the effect of the immersion in a strange culture for an unprepared visitor, Toffler characterized the future shock as the shattering stress and disorientation brought on by the premature arrival of the future (Toffler 1965). The concept of future shock has been then elaborated in the 1970 book, where it was characterized as a consequence not of the “quantity” of change but by its increasing rate: it occurs in individuals who are subjected to too much change in too short a period of time (Toffler and Toffler 1970). To the authors, the symbol of the increasing pace of change was the technological revolution, with dramatic innovations in transportation and the rising of computers as “super-machines” that were occurring in those years. However, the great engine of technology has knowledge as its fuel (p. 30), and it is to the velocity of knowledge development that the construct of future shock primarily refers. Indeed, the cognitive and psychological distress of future shock happens to be the result of an overstimulation offered by society with its technological, societal, and economical changes which cannot be processed cognitively and rationally by individuals. The authors name this condition as “information overload” that forces people “to adapt to a new life pace, to confront novel situations and [...] to process information at a far more rapid pace than was necessary in slowly-evolving societies” (p. 352). Of course, the examples of technology and the vision of future to which Toffler and Toffler referred in their work have dramatically changed since the 1970s. However, the core of their argument, i.e. the increasing pace of change of knowledge production and application, remains valid nowadays (Butler 2016), making the future shock even more dramatic than it was half a century ago.

Elaborating on the phenomenon of future shock, sociologists started to investigate its impact on human beings, identifying some sociological problems that were emerging in modern societies. In recent years, the German sociologist Hartmut Rosa coined the expression of “society of acceleration” to describe the current society and its relationship with the perception of the future (Rosa 2009, 2010, 2013). According to Rosa’s analysis, modern life is subject to three major dimensions of acceleration: (i) technical acceleration; (ii) social acceleration, i.e. changes in the institutions through which we bring order to our lives; and (iii) acceleration in the pace of life, i.e. the general sense and experience of time and deadlines on a day-to-day basis (Rosa 2010, 2013). Technical acceleration is central to Rosa’s argument and is indicated as the main driver. Indeed, in contemporary societies, humans do not often interface directly with the natural world: they live in a techno-ecology. It is such techno-ecology that, in contemporary societies, mainly drives (and accelerates) global dynamics. Another central issue of Rosa’s argument is that social institutions, like political and educational systems, despite changing, are not able to keep up with the pace of technological transformation. Because of their inertia, things seem to change very quickly, but in fact they happen without any strong sense of directionality. This perspectiveless state leads to what is called “frenetic standstill”. Events remain episodic in both individual and collective experience, and because of the lack of directionality, “nothing remains the same, but nothing essentially changes” (Rosa 2013, loc. 7100).

The impact of frenetic standstill on individuals appears as a sense of fragmentation or dispersion of the sense of self (Giddens 1991; Gee 2001; Bauman 2000; Bauman 2001). Following Rosa’s argument, identity becomes *situational*:

The individual’s reaction to social acceleration […] seems to result in a new, situational form of identity, in which the dynamism of classical modernity, characterised by a strong sense of direction (perceived as progress), is replaced by a sense of directionless, frantic motion that is in fact a form of inertia. (Rosa 2009; p.101)

On encountering such a sentence, no one, especially those who care about education, can remain indifferent. Indeed, we have all been surrounded by this imperceptible feeling of breathlessness, disorientation and disharmony caused by the coexistence of different misaligned times. On one hand, we have the time we need for reflection, taking care of ourselves and people around us, learning, figuring out solutions for pursuing social equity or addressing global challenges like climate change. On the other hand, we see the world running, innovating, competing, exchanging at an impressive velocity and creating continuously new and unpredictable scenarios and needs that leave behind all other requirements. This misalignment leads to a sense of fragmentation in our experiences that can lead to frustration but also forms of burnout.

To describe this fragmented and meaningless perception of happenings, Rosa refers to the distinction between two German terms that, as Walter Benjamin recognized, can be used to talk about episodes of present: *Erlebnissen* and *Erfahrungen*. Erlebnissen are episodes of mere experience. When we live through them, they seem never-ending and leave no trace in the memory. Because of this feature, Rosa calls them *long-short* experiences. Erfahrungen are instead experiences that leave a mark and contribute to building our identity. When we live through them, they seem to pass very quickly but leave a trace in the memory. Rosa calls them *short-long* experiences. A century ago, Benjamin complained with some concern that we were approaching an era rich in Erlebnissen and poor in Erfahrungen. Rosa stresses that today we are experiencing a world of *short-short* episodes (Rosa 2010). Time seems to escape from both the sides: it goes fast, and it does not leave traces in the memory. Consequently, the episodes of present remain *alien*. We are not able to appropriate them and make them “ours”, in the sense we are not able to turn them into events that are significant for us. This is the phenomenon that Rosa calls “alienation from time”. Alienation is another central concept in Rosa’s view and, following Marx, it indicates that state in which people pursue objectives and follow practices that no external agent or factor oblige them to do but they do not feel like doing or want to do (Rosa 2010). *Alienation from time*, Rosa continues, is strictly interrelated with *alienation from others* and, as we saw, *alienation from self*. In fact, our identity and sense of self derive from our actions, experiences, and relations and from how we situate them and ourselves in the social and material world and in their space-time fabric. Frenetic standstill, the sense of directionlessness, situational identity, alienation from time, and contraction of the present are all manifestations of the future shock that acceleration produces.

Struggling with the future in the society of acceleration and uncertainty has important educational implications since it is one of the main issues for the young generation. The youth perceive the future as a threat, instead of a promise (Benasayag and Schmit 2006). Without future horizons, the present is lived as completely oriented toward seizing the moment, sniffing out every opportunity and keeping open all possible scenarios: the present appears fragmented in a plurality of events that have no real temporal connections between them (Leccardi 2009).

Alienation from time and difficulties in young people in projecting themselves in their future are confirmed and illustrated in a 2015 Eurobarometer survey (Eurobarometer 2015). The educational literature on students’ perception of future has been discovering an important nuance in such perception (e.g. Cook 2016; Heikkilä et al. 2017), a sort of “duality in futures thinking: the young may see their personal future as positive and in their own hands, but they view the national and especially the global future as hopeless, frightening and completely outside their influence” (Levrini et al. 2020b). To face these issues, educational authoritative reports highlight the need to search for teaching approaches that enable to develop foresight and anticipation skills. For example, OECD elaborated an educational framework (2018) based on the *AAR (anticipation, action, reflection) cycle*. AAR cycle implements the core idea that responsible actions emerge through anticipatory and reflective processes. In fact, AAR cycle is an iterative learning process “whereby learners continuously improve their thinking and act intentionally and responsibly, moving over time towards long-term goals that contribute to collective well-being” (OECD 2018, p.2). In the cycle, the first phase, *anticipation*, is related to foresight skills, since anticipation implies that the learners exploit their abilities to anticipate the short- and long-term consequences of actions, understand their own and others’ the intentions and widen their own and others’ perspectives. The second phase, *action,* implies that learners take action in the present. *Reflection* is the third phase in which learners improve their thinking in order to reach a deeper understanding and better actions toward well-being. These competencies, if combined in cycle, are supposed to develop faster both agency and transformative competencies. 

The goal to search for a future-oriented approach is an emergent theme in science education, although still underexplored. Science education is interested in foresight for several reasons. Firstly, future views, being utopian or dystopian, are connected to science and technology (Carter and Smith 2003). Secondly, science education, more and more explicitly, can become a very locus to promote “Erfahrungen” and guide students to appropriate their time and develop the sense of self (Levrini et al. 2015; Levrini et al. 2018;  Kapon et al. 2018). Thirdly, many foresight or future thinking skills that are needed to navigate the society of acceleration and uncertainty can be easily related to scientific thinking and developed through the learning of science. We are referring to foresight or future thinking skills such as scenario thinking, systemic thinking, thinking beyond the realm of possibilities, action competence, managing uncertainty and complexity and creative thinking (Branchetti et al. 2018; Levrini et al. 2019b; Tasquier et al. 2019).

The incorporation of STEM activities in the curricula that could foster the development of foresight or future thinking skills is the main goal of the two extensive projects in science education. The first project has been carried out in Australia by Paige and Lloyd (2016) who developed an educational approach that integrates a future dimension into science learning to enable students to develop a broader future-oriented perspective that can impact on many aspects of their lives. They stress the necessity to identify and envision alternative futures that are more socially and environmentally fair and sustainable. The second project has been developed in Europe. The research group of Physics Education of the University of Bologna, together with colleagues from the University of Helsinki and an Icelandic NGO, studied how school science can equip and empower secondary school students (14–19 years old) to face uncertainty and contribute meaningfully to their own and global futures. They coined and operatively defined the concept of “future-scaffolding skills” that are skills to construct visions of the future that support possible ways of acting in the present with an eye on the horizon (Branchetti et al. 2018; Levrini et al. 2019b; Tasquier et al. 2019). Most of this study has been framed within the Erasmus+ European project I SEE—Inclusive STEM Education to Enhance—the capacity to aspire and imagine future careers (www.iseeproject.eu). The educational approach is described extensively in  Levrini et al. (2019b, 2020b), Tasquier et al. (2019) and  Branchetti et al. (2018). 

As an outcome of the project, four teaching-learning modules were designed and tested. Every module was built on a different “future-oriented scientific issue”: a scientific and future-relevant topic  which is at the core of current societal debates and may be of interest to students. The topics of the four modules were climate change, artificial intelligence, carbon sequestration, and quantum computing. The modules were designed on the assumption that science is an impressive source of knowledge for thinking and talking about the futures (Levrini et al. 2019b). Indeed, the epistemic practice of building predictive models and causal structures for explaining and foreseeing natural or social phenomena is constitutive of science. Science has developed various models of causality. As well as the linear and deterministic models of Newtonian mechanics, twentieth century science has elaborated more and more sophisticated probabilistic models (Tasquier et al. 2016). Quantum physics and the science of complex systems are rich sources of concepts such as space of possibilities, future scenarios, projection instead of prediction, uncertainty, agent, feedback, and circular causality, which can be suitable for development into skills for thinking and talking about the future (Levrini et al. 2019b) as well as for developing citizenship skills (Barelli et al. 2018).

The implementation of the I SEE modules was monitored and led the researchers to progressively identify, define and refine operational markers able to (i) describe the change in students’ perception of the future through the modules and (ii) identify future-scaffolding skills (Levrini et al. 2019b; Tasquier et al. 2019; Levrini et al. 2020b). These studies pushed us to ask how the teenagers were experiencing the period of lockdown and isolation. Within I SEE, we touched upon the great difficulties encountered by young people in imagining their futures, and we closely touched how challenging and demanding it was to guide them to develop future-scaffolding skills. As a follow-up of I SEE, we aimed to investigate if and how students’ perception of time was influenced by the quick changes induced by the pandemic and to create a new baseline to reflect on the role of science education in supporting the young as they navigate through fast-changing space-time structures.

### “Present Shock” and Time Re-appropriation

The COVID-19 pandemic, besides many aspects of our lives, has deeply transformed the time structure that regulates our social and individual rhythms. This situation shows aspects that are different from those that characterize the society of acceleration. First, acceleration has, during pandemic, a natural origin, and because of this, its pace of change is a matter of fact: nature is following its own evolution. All our cultural systems have been following the virus diffusion and are searching to identify its biological, epidemiological, and clinical features in order to slow down the pandemic contagion and save the health systems and societies. Available scientific, mathematical, and technological knowledge has been applied, and the production of new knowledge and technology has been accelerated as far as possible. When a pandemic is occurring, just like now, science and technology are chasing behind the natural evolution of the virus in order to produce a vaccine and to elaborate recommendations for governments, policymakers, and other institutional panels. Science and technology are fighting against time and many forms of uncertainty. For example, even if international guidelines for data reporting and sharing have been developed even before the COVID-19 pandemic (Modjarrad et al. 2016), science is fighting against the lack of national and international standards for data collection and sharing (Lau et al. 2020), against the overproduction of fake news or pseudo-scientific rumours that continue to influence people (Frenkel et al. 2020; Bavel et al. 2020) and, in many countries, against the slashing of resources in research and public health systems (Bong et al. 2020). Also, methods of interaction (in an official, reliable, and trustful manner) with policymakers take time. This race by science shows the dramatic dilemma for scientists, who have to balance the urgency of a situation requiring immediate responses with the time required by scientific methods, which is intrinsically longer (the time to collect reliable data, analyse them, elaborate models and hypotheses to test and revise, the time for peer reviewing and the time to move from foundational research to the production of drugs or vaccines). Regarding policymaking, governments and municipalities are required to make immediate decisions in conditions of emergency to “flatten the curve” and try to limit the risk of collapse in the social and health systems. Even in the most virtuous cases, we have observed policymakers and governments who were reluctant to learn immediately from precedent experiences (e.g. from China, South Korea and Italy) (Balogun 2020). Part of the problem is that each government is focused to take into account also local political and cultural peculiarities, according to which decisions are based on different priorities or values (Weible et al. 2020), as well as on different conceptions of risk (Greco 2020). When decisions have to be taken so quickly, the risk of asymmetric effects that favour some categories over others is very high. The search for an equilibrium between conflicting interests, objectives, subjects, institutions and their various dimensions (health, economic, psychological, societal, educational, etc.) takes time, but this time is also the ground of democratic systems and social cohesion.

In many countries all over the world, people have been requested to lock down and stay isolated and act as responsible citizens. From our houses, we have been eyewitnesses to a worldwide phenomenon, and under a careful look, dynamics and practices that are usually invisible can be discovered in such special social periods. We can see and learn, for example, how science works, how our societies are organized, how our institutions and leaders operate and what values (economy, health, public wellness, solidarity or private interests) have been prioritized by our leaders. We can also learn about the role ascribed to science and technology in our social contexts and what role is designated to knowledge and education.

However, whilst we have the chance to observe these complex structures emerge, our daily time appears suspended. We are searching for new rituals, new habits and new priorities that can normalize and control the time of our daily practices. Thus, the time structure of a society during pandemic is rather special. Outside, the public and social world is running as fast as possible to contain a disaster; inside, in the places where lockdown was disposed, the private and daily world is grappling with a new form of slowness. The society of acceleration, Leccardi argues, has reduced the time of everyday life to trivial and repetitive rituals that have slowly disappeared from the attention of our societies (Leccardi 2003). This time, instead, includes a precious embodied dimension where time is linked to concrete spaces and is made up of gestures and acts that follow multispeed rhythms. In daily lifetime, the private and public world can be kept together (Leccardi 2003), and in the risky, fragile and global world, the future of our societies and the planet depends increasingly on everyday rituals (Beck 2000). In Italy, lockdown, at national level, lasted about 2 months (from March 9 to May 4, 2020), and during that moment, we felt the urgency to investigate how teenagers were experiencing their time.

As we will describe in detail in the methodology section, the match between the data we were collecting and the feeling that we were perceiving during lockdown led us progressively to formulate the following hypothesis: the lockdown period led people and students, in particular, to the rediscovery of daily time and the recognition of its enormous value also as a locus to take agency and build empowering visions of the future. Compared with the precrisis period, the sense of alienation from time gave way to the feeling of time re-appropriation. We were all re-appropriating the meaning of our daily rituals and perceiving a new sense of directionality: although the future was still invisible, we perceived that we are witnessing an epochal transition. We called this general feeling “present shock”, and we decided to carry out a survey aimed at testing such a hypothesis, that is, testing whether students were really re-appropriating their time and how such re-appropriation looked like.

*Appropriation* is a key word in the learning sciences and in science education. When used in these fields, the term is borrowed from scholars in linguistics and education (Bakhtin 1981; Rogoff 1995) and used to link disciplinary learning with a special personal engagement that makes learning authentic (Kapon et al. 2018). Bakhtin in linguistics defines appropriation as follows:

It [a word] becomes ‘one’s own’ only when the speaker populates it with his own intentions, his own accent, when he appropriates the word, adapting it to his own semantic and expressive intention. Prior to this moment of appropriation, the word does not exist in a neutral and impersonal language (it is not, after all, out of a dictionary that the speaker gets his words!), but rather it exists in other people’s mouths, in other people’s contexts, serving other people’s intentions: it is from there that one must take the word, and make it one’s own. (Bakhtin 1981, pp.293-4)

When we apply the quotation to science learning and refer appropriation to a concept like force, temperature or entropy, appropriation implies productive learning including deep conceptual understanding, but it also involves a reflexive process of populating scientific discourse with personal intentions, purposes, and tastes that allow a student to embody scientific discourse and concepts in a way that is authentic and personal. This usage of the term appropriation stands in explicit contrast to the more typical connotation of “appropriation of scientific discourse” that would imply that students learn to “speak like scientists” or use words in the formal register of physics without necessarily developing personal meaning. On the contrary, when students appropriate a concept, they begin to speak an “authentic” language, which has meaning and relevance for themselves as well as being respectful of consolidated knowledge (Levrini et al. 2015).

The construct of appropriation has a long history in the research literature, tracing back to Vygotsky (1978). After Bakhtin (1981), it was use and developed, for example, by Rogoff (1995), Sfard and Prusak (2005) and Sfard (2007). In science education, it has been operationalised as a theoretical construct in a study that concerned secondary students’ reactions to an extended teaching intervention on thermodynamics (Levrini et al. 2015, 2018). The analysis of data collected during that implementation demonstrated how students not only made progress in learning the disciplinary content of the teaching/learning path but also made progress making the material relevant in a personal sense. A bottom-up, fine-grained qualitative analysis of students’ interviews and classroom discussions allowed the authors to identify five discursive markers for operatively deciding whether students had appropriated the scientific discourse of the unit (Levrini et al. 2015). The discourse of students who spoke their own language and revealed appropriation presented the following features, being:

A *Developed around a set of words or expressions* repeated several times and linked together so as to express a *personal*, *idiosyncratic signature idea* with respect to a physics topic (thermodynamics in that case)
B *Grounded in the discipline*, i.e. the signature idea was used by the student to understand scientific concepts in the sense that it was used as a tool for focusing on pieces of disciplinary knowledge, selecting and re-assembling them to make sense of the specific concept (e.g. temperature)
C *Thick*, i.e. the signature idea involved both a metacognitive dimension (showing what learning physics means for the student) and an epistemological one (showing what sense of the discipline the student has)
D *Non-incidental*, i.e. the signature idea was expressed in several activities throughout the students’ classroom experience, not just in one interaction or in the moment of the interview
E *Carrier of social relationships*, i.e. the signature idea allowed the student to play a specific role in class discussions, and this role was acknowledged by others in the classroom community

The markers indicate that appropriation is a multilayered process where the students take an idea and elaborate it according to their own interests and testes (marker A) and coherently use the idea to position themselves with respect to disciplinary knowledge (marker B), with respect to others (marker E) and with respect to their own personal story of who they are as a person and a learner (markers C, D).

In the next sections, we show how the markers of appropriation inspired a way to investigate how teenagers are now grappling with the present shock and whether they are searching for strategies to appropriate their time during lockdown. The survey will show that the markers were helpful for outlining articulated profiles of time management. However, in order to unpack the relevance of the markers, we needed to refine the structure of the appropriation markers with further sub-markers coming from the literature in sociology of time that we introduced above. In particular we needed to interpret the personal conceptions of time that emerged in terms of their relations with (i) daily life rituals, (ii) the sense of agency on ones’ own time and (iii) the sense of time directionality.

Such sub-markers allowed us to stress that, whilst the alienation from time leads to a sense of identity fragmentation and identity to be situational, time re-appropriation, during lockdown, leads to a re-appropriation of the self and of the others, that is, a re-appropriation of the sense of what diSessa calls “intrinsic identity”: that form of identity that “concerns patterns inherent in our varied and distinctive ways of interacting with the world, including the social world.” (diSessa 2018). This is not surprising since, in moments of deep change, we are all required to deal with the need to realign our self-perception as agent or receptors of change, producers of sense-making, as well as the need to refine our norms and criteria to manage ourselves with respect to others. In this sense, identity is at issue. The link between appropriation and identity is widely described elsewhere (Levrini et al. 2018). Here, we simply stress that when, in our analysis of students’ discourse, we saw terms and expressions regularly repeated by the students (marker D) that appeared idiosyncratic (marker A) and thick (marker C), then we assumed that those pieces of knowledge mirror aspects that can be related to intrinsic identity.

## Methodology

The study was carried out to answer the following questions:

a How are secondary students experiencing time and what, if any, knowledge are they using to grapple with the changes in its pace?
b How does the learning of science contribute to supporting the students to rearrange their daily lifetime rituals? More specifically, does science learning support students to deal with global concerns such as pandemics and climate emergency and to grapple with quick changes in the time structures of our complex and fragile risk society?

A survey was designed with an exploratory and piloting nature. It was not meant to provide comprehensive answers but to pave the way and lay the foundations for further, wider and more focused, rounds of investigation.

For this exploratory survey, we chose the tool of extensive individual interviews and a qualitative approach for the analysis. We then decided to focus on a special group of secondary school students. We selected students at the end of their secondary studies (17–19 years old, grade 12–13), close to making their own university choices and particularly interested in science (a scientific degree course was seen as one possible option for their future university career). The students that we involved in the survey had attended extra-curricular courses on advanced STEM topics (quantum computers and climate changes), organized by our Department, in January and February 2020. We chose these students to maximize the probability of encountering teenagers who refer to or use scientific knowledge and competences to deal with the current changes. In this sense, we considered them particularly suited for “breaking the ice,” hewing the theme and providing tips to refine tools and aims for further stages of the research. 

Nine students (4 female and 5 male) agreed to be interviewed. They were all attending the same type of school, a “Liceo Scientifico”, scientific-oriented secondary schools in Italy. But they did not know each other, since they were from different schools which were also in some cases in different cities.

The protocol of the interview is reported in the Annex. It includes 23 questions, articulated in two main sections: in two main sections: (a) Section 1: Questions about the ways they were experiencing the lockdown (b) Section 2: Questions about their perception of time

In the first section, the questions concern the changes they perceive in their everyday life, the role they ascribe to school and school science, the role of science they see in the current social and political debates, the channels of information they use, the kind of individuals they consider experts and the type of trust they feel. In the second section, the questions regard (a) the role played by past, present and future in their thinking when they have to deal with difficulties and/or to make personal choices; (b) their perception of time in the present daily life; (c) their perception of the future in the short-, medium- and long-term period; and (d) their perception of the scale of the pandemic in the “big” history and its effects on social transformations.

We used three main criteria for the specific formulation of the questions:

- The questions had to reveal, where existing, future-scaffolding skills.
- The questions had to foster a relaxing interaction aimed at development of thinking and generating knowledge in the interaction.
- The questions had to make the interview a significant moment, during which the students were guided to elaborate their own experience, discover possible ways to grapple with its novelty and participate in the social debates.

The interviews were conducted in videoconference by OL, SS and GT. Each of them lasted between 1 h and 75′. They were produced during the lockdown period in Italy, from late March to early April 2020. The interviews were audio-recorded and transcribed.

The data was analysed through methods inspired by grounded theory (Anfara et al. 2002; Glaser and Strauss 1967), which we generally apply in the analysis of extensive interviews or classroom data (Levrini et al. 2015, 2019a). The analysis was performed through explicit back and forth dynamics, from data to their theory-oriented interpretation. In accordance with our second research question, the interpretation is also oriented toward concrete support for teaching. This combination of bottom-up and top-down processes, from data to theoretical interpretation and vice versa, was carried out through a series of systematic de-briefing and triangulation meetings among all the authors of this paper. The overall process comprised four phases.

### Overview of Data

The first phase corresponds to an overview of the whole corpus of data to identify a sensitizing idea (Glaser and Strauss 1967) that could guide us in data immersion. The compass we initially used to navigate the corpus was inspired by the notion of “future shock”. We examined the data by identifying which pictures of the future emerged and looking for analogies between the future-scaffolding skills and the skills that the students declared or revealed for managing such a period of emergency. At the beginning, this phase was very frustrating since the answers to the questions on the future were the poorest part of the interview, and the way students talked about their strategies and feelings in this period were very weakly linked to the categories we had elaborated and refined in our previous experiences. They either mirrored a general tendency to deny thinking about the future (“I do not see any differences in my way of perceiving the future”, “I remained pretty much myself”) or reproduced the well-known distinction between a concrete and positive image of a personal future and an uncertain one. Sentences like “this pandemic will finish and I will go back to my normal life”, “my plans have not changed, I am just thinking about ways to get through this period as well as possible” or “I’m looking forward to starting university and getting on with my life” were very frequent.

It soon appeared clear that the pandemic did not act significantly at that level and that the future was not the most interesting theme or time dimension of the interviews. Although we found this result initially surprising, we then realized that it was coherent with the sociology of time and with our hypothesis that the pandemic acts more on perception of the present. In particular, the most satisfying moment of the first phase was the discovery that, maybe, students were grappling with the issue of appropriating or re-appropriating their own present time. Starting from this intuition, we fleshed out, as a *sensitizing idea*, the hypothesis that our appropriation markers could be revised and used to capture how students were grappling with time and, in particular, how they were trying to stay consciously in the present of their current experiences. This intuition moved us forward to the second stage of the analysis.

### Construction of a Shared Grid for Data Analyses

The second phase corresponds to the elaboration of the analytical grid. We started from our appropriation markers and revised them so as to fit with the theme of the interviews. The change of context, from appropriating a physical concept to appropriating time, naturally required a significant adaptation of the five markers which were then transformed into the following sets of questions, used in the interview:

a Is there an idiosyncratic idea about time management which the students focused on in the interview (what terms, expressions, etc.)?
b Does the student use disciplinary/school knowledge (particularly scientific knowledge) to manage their time (grounded in the disciplines)?
c Is the core idea thick, i.e. is the student able to develop her/his core idea in a consistent way throughout the interview? Does she/he appear aware of it?
d Is the idea non-accidental, i.e. does the interview suggest that the core idea is grounded on experiences and reflections that are not only suggested by the contingency of the interview?
e Is the idea a carrier of social relations and/or does it allow the student to see her/himself situated in society?

Two further sets of questions have been formulated with the explicit aim of reminding us to remain focused on the theoretical orientation of the analysis and the relevance of the results for teaching:

a What contribution does the interview make to help refine the idea of present shock and to build a baseline framework on students’ strategies to managing fast-changing space and time structures? Do a sense of slowness, directionality and the importance of daily rituals emerge? Do they see their role in social cohesion and as agents of history?
b What contribution does the interview make to help us build an argument about the role of science education and the role of disciplinary knowledge to equip the students with knowledge and competences to manage fast-changing space and time structures?

The questions have been slightly reformulated throughout the process of analysis to make them significant for all the interviews and effective in capturing the most interesting points of the students’ discourse.

### Immersion in the Data and Profile Construction

The third phase was the core of the analysis, represented by full immersion in the data. The application of the analytical grid allowed us to outline students’ profiles. Six complete profiles were produced by selecting the most different interviews. Three interviews have been used as sources to comment and discuss the results.

Before applying the grid to the whole corpus, we started with two interviews to check whether the questions made sense and were easy to apply. To make this check, at least two people, independently of one another, listened to the interviews and highlighted the most interesting excerpts directly on the transcripts (pure bottom-up process). Then, two or three researchers confronted the highlighted sections, and having agreed on the selection, the sentences were compared with the questions and positioned under the question they were judged to best answer. We quickly realized that the set of questions was effective in capturing all the highlights and for organizing them in an apparently faithful profile of the student. At least two researchers worked on each “profile”. When all the profiles had been drafted, we realized that the initial formulation of the questions left room for different interpretations, and this compromised the comparability of the profiles. We then organized an alignment meeting to share an increasingly operational elaboration of the questions.

The meeting resulted in the following list of instructions:

A To search for the *idiosyncratic idea*, check if there are words that the student repeats more frequently to talk about her/his way to manage time, which cannot be ascribed to the interviewer. If so, select the core sentences where the keywords are structural and try to paraphrase the core idea with a catchy sentence that can help transmit the essence of the idea. As a final step, select from the interview all the excerpts that show if and how the idea has been elaborated throughout the interview.
B In order to check whether the core idea is *related to disciplinary/school knowledge* (in particular scientific knowledge), focus on all the parts of the interviews where the students talk about subject matters or science. Distinguish between the sentences where she/he mentions contents, the sentences where epistemological or methodological aspects are used or mentioned and, finally, sentences that refer to the theme of science and society and/or to the role of experts.
C In order to check the *thickness of the concept*, search for its consistency within the interview and, where possible, its nuances and articulation throughout the interview. We are making the hypothesis that inner consistency of the discourse can reveal a form of awareness and depth of thinking.
D Consider that accidentality refers to the fact that students’ discourse does not touch upon identity aspects but has arisen spontaneously under the stimulus of the interview questions. Thus, in order to check and “measure” *non-accidentality*, check if the student mentions or refers to experiences and thoughts from outside of the interviews. Be careful to remember that we are all experiencing an extraordinary period. Thus, distinguish, in the interview, between two types of accidentality/contingency that the student can show: the accidentality of the interview (is she/he inventing a position just to manage an issue, contingently raised by the interview?) and the contingency of the pandemic (is she/he reporting about an issue that she/he had never addressed before, because of the novelty of the COVID-19 crisis?)
E In order to check if the core idea plays any role to *position the student in society*, describe the social network mentioned by the student (if she/he refers to friends, family or society more on general) and describe the eventual links between the core idea and the type of interpersonal relations and social positions in which she/he feels immersed now or in the future.

On the basis of this operational grid, we finalized the extended profiles, which can be downloaded here.

### Double Check of the Profiles and Results

Once we had all the profiles, we compared them first with each other and then against our theoretical framework. The comparison was carried out along three axes:

i The pictures of time that emerged from the interviews
j Appropriation, its nexus with identity and its contribution to defining present shock
k The role attached by the students to science and its learning

Regarding the first point, we operationally focused mainly on the first part of the profiles (the core idea that emerged by applying marker A). As for the appropriation analysis, we operationally referred to a summary on the five markers application, as well as to the first extra question, which concerned specifically the contribution of the appropriation analysis to the sense of present. In order to unpack the appropriation markers, we applied three further criteria coming from the sociological literature (the role of daily life rituals, the sense of agency and directionality).

Finally, in order to compare the roles that science knowledge and education played in students’ approach to managing the crisis, we operationally focused mainly on the second part of the profiles (the results of the application of marker B), as well as on the last extra question. From there, we identified indicators for the next agenda for science education.

The results from this phase are presented and discussed in the following sections.

## Results

### Students Conceptions of Time in the Pandemic Era

The first result concerns the emergence of an articulated and stratified picture of students’ perceptions of time. From the profiles, three main conceptions of time can be recognized: (i) time as an opportunity to immerse yourself in experiences (4 students), (ii) time as agenda (1 student) and (iii) time as an empty container to fill (1 student). We use this macrostructure to present the students and sum up the core idea that they expressed in their interviews. In order to protect anonymity, students are identified by pseudonyms, but gender distinction is maintained.

#### Returned Time as an Opportunity to Immerse Yourself in Experiences (Chiara, Alessandro, Gabriele, Laura)

Four of the six students in question see the current time as an opportunity to immerse themselves in their experiences.

Let us start with Chiara. The core idea of time that emerged from her interview is:

In the motionless and suspended time created by the pandemic, one can find ways to manage the oppression of the present with new activities to control time and by escaping with the imagination toward fantasy scenarios.

Chiara’s whole interview revolves around her perception of “velocity” of time. Before the coronavirus crisis, she was “really full of things to do, time passed very fast”. Now the time has slowed down and “it is much slower, actually, it is also more difficult to face [...]”, she feels that “time is a little suspended”, she “never really feels it moving”. The stillness of the present is coupled with a sense of anxiety caused by everyone around her talking about the pandemic: “I feel very surrounded by all this. The news every day, my mom, my dad, my friends who talk about it”. She perceives strong differences between her personal time and the time of society from which she feels detached: “they all move super-fast, actually, they all seem much more on it [...] everything outside happens very fast”.

Chiara has found two main ways to manage her motionless and oppressive present state. Firstly, she enriches her daily routine with a lot of new activities (cooking, playing the piano, training, video-calling friends). From the interview, it is clear that these activities are not only new rituals with which she fills the empty container of her time but new distractions to control time. The other way she has found to manage the oppression of the contingency is mental escape, which makes the present lighter and more liveable. She often loses herself in a fictional world in which she is not surrounded by reality, and this is not only something she likes but something that really helps her: “Also watching movies... in this moment it’s nice to see the dystopian ones or science fiction [...] it helped me a lot to have read, to have seen some apocalyptic films”.

As well as Chiara, Alessandro expressed, in his interview, the idea that the pandemic has allowed him to recover time which, free from external commitments, is a “time given back”. For Alessandro, this time is a great opportunity to immerse himself in personal intellectual experiences. Moreover, it is also a good opportunity to look for new ideas for reflection on social issues with two goals: to acquire new knowledge, but also, and mainly to broaden his own “points of view”. Alessandro is deeply aware that building thinking tools is not an immediate and easy process. It takes time.

The organization of the day must certainly consider school commitments, relaxing activities and social conversations with friends, but above all there is room for “that free time”, which is no longer a “counted time”. There is the possibility to choose how to use these “slices” of one’s time, and for Alessandro, it means having the possibility to immerse himself in personal intellectual experiences: latent intellectual interests, such as an interest in philosophical thought, could re-emerge and be developed. The “returned” time is therefore not the counted time of the clock, but a mental time, the time necessary to cultivate “that thought”—free from other thoughts.

It re-emerged in this period [the interest in philosophical thought], I already had it, I just didn't have “slices” of free time, and [...] now it's coming out quite disruptively, [...] I'm glad because there are all the conditions necessary to bring out the interest. […] Now I can “slice” me up some time and dedicate myself only to that and I like it even more because I can immerse myself in it ....

“Returned/liberated time” is that time “to stop and rethink oneself” both as regards the personal dimension linked to one’s own interests, dreams and expectations for the future but also as regards the social and political sphere.

The third student, Gabriele, also took this moment as an opportunity to immerse himself in his thoughts. In the interview, he expresses the idea that the “right way” to spend his restored time is looking for the essence and the truth of what is happening, looking at history as a source of knowledge about “mankind”, the “invariant”, “what does not change”, in order to pursue progress and improve the human condition. He refers to two kinds of time: internal (personal) time, to be filled in the right way (“you have to find the right way to occupy time”) with passions, reflections and trying to find the “right non-routine routine”, and external time, the “time of mankind”, which is linear, deterministic, concerning “what lasts”, “what does not change” (“... man is always the same, man always does the same things ... technology changes, changes ... there is incredible progress but in the end it is always man, it is always him ...”).

From what he perceives as a privileged position, Gabriele observes that this pandemic period removes the veil of Maya, obliging people to become aware of all the critical issues and difficulties that have always existed:

The virus simply showed [the problems we are observing on a social level], but we were already in a relative moment of crisis ... [...] But it has always been like that ... that is, there have always been... simply that before they were directed toward different topics, [...] the virus brought nothing new except this state of quarantine.

It does not create any sense of upset in Gabriele, since he experiences the crisis as something “outside of him” (“really the concept of quarantine itself, of this ... of this virus ... I don’t feel it ... it’s not inside me ...”). The “right way” to spend time is, therefore, exploring the surrounding world by searching for the essential, by finding invariants that are not scratched by time.

Then we have Laura. Like Alessandro, Laura describes time as a personal non-routine construction, which has to be invented and re-invented by fitting together as many activities as possible: “the better I can fit things together, the better I can manage them”.

The pandemic caused a “drastic change” in her daily life organization: from rhythmic days organized around extra-school and outside-home commitments to the problem of finding other activities to fill the time. The quick change imposed by the pandemic led her to lose, at the beginning, any sense and conception of time: “I’m mostly losing the concept of time, completely ... that is, not what day it is today ... for example, I don’t have the perception of when it was a week ago ...” After an initial moment of great bewilderment, the awareness that it was necessary to react emerged. At first it seemed like resignation, and then she found a way to rearrange her time by allowing old passions to re-emerge, like playing music, reading novels and mastery of astronomical topics. The implications of the drastic change that she complains most about regard the fact that many out-of-school commitments, like a course on particle physics at CERN planned for the immediate future, have been cancelled. They represented “certain moments”, sort of milestones, in her path toward the choice of university. The solution she found to replace these activities is to read and study the syllabus of university curricula and try to imagine herself, in the future, attending those courses: “I think yes, it gave me confidence and certainly comfort to see what I could do, I have to say that looking at the degree courses or things like that is making me excited, so ...” All these aspects show that time, for Laura, is neither something awarded by external or social constraints nor an empty container to fill, but a personal construction built by fitting and cultivating personal interests and passions.

#### Time as Agenda (Giorgia)

One student, Giorgia, describes her way of grappling with time, which is rather different from the students we have discussed so far. Giorgia’s interview revolves around the following core idea on time: “I have to try to understand how to manage time better and try to take advantage of this time that now I have”*.* Time, for Giorgia, emerges as a personal agenda that she creates in accordance with external commitments to optimize her personal path and pursue her goals. Lockdown is seen as an opportunity to have empty slots that must be capitalized upon. She feels as though she is in a crucial moment of her life where she has to choose the university degree course and she feels the pressure of this choice: “especially in this period I have to think about my university choice and it’s not easy because, anyway, maybe I choose the wrong option; that is, it seems that if I make this [wrong] choice, this will be my future”.

Giorgia is very focused on adhering to her daily routines, whilst she does not follow any political, sanitary, or public debate. School and homework, Instagram, YouTube, and apps to chat with friends help her to routinize very concrete activities (e.g. learning to edit videos). Youtubers and influencers also help her to “accept also empty times”: “[There is] a woman, a Youtuber, who has [been] a kind of comfort because [...] She said that it is normal that in this situation there are moments when, in fact, you would not feel like doing anything”.

The core idea appears strictly related to her self-confidence and trust that, if she focuses on her training and if she works hard, she will be able to reach her goal to achieve a leadership position: “I always imagined myself, I don’t know in what area, I don’t know how, I don’t know where, but that I was able to reach a high level in what I was doing. [...] I have always seen myself, since I was a child, perhaps very probably also very influenced by the films I saw and various things, just ‘I who enter my office as a boss, manager of what I’m doing.’ So, my job, my great goal is to be able to reach that level...”

A similar position is expressed by Caterina and Dario, two of the three students we interviewed without outlining the profiles. Caterina and Dario are also very focused on their education, and in addition to capitalizing on the extra slots to improve their calculus skills in order to become researchers in physics, they are also taking the opportunity to learn to strengthen their “resilience” (Caterina) or concentration (Dario) skills.

#### Time as Container (Stefano)

Only one student, Stefano, expressed the implicit idea that time is a container that must be managed by filling it with engaging and enjoyable activities. Daily time is perceived as an ordered, rhythmic organization of school commitments and social activities with friends. Stefano is particularly interested in scientific subjects (physics and mathematics), and his identity is strongly related to his being a very good student, extremely talented in physics. Because of this time perception, the COVID-19 crisis did not create a deep sense of disturbance: his world and his temporal organization are still, as before the crisis, populated by rites of daily life that involve (above all) his school commitments, the study of physics and video-chatting with friends—this activity is aimed both at sharing leisure time and helping each other in scientific disciplines and any technological issues related to distance learning. The main change in his time management produced by the COVID-19 crisis concerns a sense of emptiness and a sense of urgency. In particular, regarding the sense of urgency, he stresses that the crisis showed that time should not be wasted, and priorities can be changed:

The priority of things has changed; if before I did A, B and C now I have realized that C is more important and therefore I dedicate more time and, above all, more energy. Before I would say ‘okay, I’ll do it tomorrow’ - now it's not tomorrow, I have to do it now.

The rituals and hobbies allow him to feel protected from the external world and external suffering. He does not follow any social debate since he finds them painful and he and his friends prefer to take the situation more lightly:

We take the situation as a game, but not a game intended as fun; also because according to Instagram there are many jokes, a lot of jokes about this situation that is happening, probably I also didn't really understand what it is and therefore we don’t feel this great need to say "oh my God, this is happening", [...] so we don't feel this great need to remember it even when we are together [with friends], when we are together it should be a moment of relaxation …

#### Comparison of Profiles

The three-pronged picture of time perceptions that emerged from the profile (time as container, time as agenda, time as personal time of immersion in the experiences) mirrors the debate between *substantivalism* and *relationalism* that can be traced back in the history and philosophy of physics (e.g. Huggett and Hoefer 2018). The distinction of space-time as containers or as relations, indeed, seems to capture the two more general ways that people use to think of and to talk about time. In students’ conceptions, we can recognize, in the words of Stefano, the echo of the Newtonian conception of time as a container that provides an external organization to the multiplicity of the events. All the other students appear to be agents of their time, and their words echo the view of space and time as a set of relations: a social or personal construction, elaborated to organize experiences and provide them with “an order of succession” (Leibniz, 1715–16 in Alexander, 1956, Third Paper, paragraph 4; G VII.363/Alexander 25–26). From our bottom-up analysis, we found two different ways to conceive the relations that create the sense of time: “an order of succession” to be ascribed to activities and organize daily time according to that *agenda* and a time generated by *immersive experiences in personal thoughts*. The first is the case of Giorgia, whose time is created by planning her activities so as to capitalize on experiences and select the most relevant for her future. For the other four students (Chiara, Alessandro, Gabriele, and Laura), the sense of time is created by immersion into personal reflections that can involve different plans: imaginative (Chiara), socio-political (Alessandro and Gabriele) and intellectual (Alessandro and Laura).

This last remark highlights the fact that the overall picture emerging from students’ profiles is more nuanced than the three-pronged macrostructure we fleshed out. Such richness needs a different lens in order to be captured. In the next section, it will be inspected through the lens of appropriation that will zoom in on the nexus between time perception and identity.

### Appropriation of Time and Its Nexus with Identity

After the presentation of students’ conceptions of time, we can now focus on the *appropriation profiles* that emerged from the application of appropriation markers as lenses to read the transcripts. The most important result is that the application of markers resulted in five complete profiles of appropriation and one incomplete profile: all the markers were satisfied for five students out of six. Indeed, only Stefano, the student who had the picture of time as a container, did not appear as an aware agent of his time and encountered a little difficulty in the metacognitive or reflective questions. His interview indeed looks like a collection/description of small activities or examples referring to his private life. He never answers a question at a level of meta-reflection and never refers to abstract concepts to reflect on time and the effects of this crisis. Instead, in his answers, he always refers to local examples or contingent cases related to his families and friends. For example, when he is asked to answer this question: *are you currently feeling pressure or are you experiencing accelerated times coming from outside?,* he answers as follows: *then the most important thing concerns perhaps the work of my parents who, however, since ... [said slowly] that is, the work of my parents ... the work in general.*

Stefano is also the student who least perceived any change. His daily rituals created a bubble that protected him from the external world, and in this bubble, there is neither directionality nor any relationship with the accelerated time of social institutions that are running after the virus and trying to control it. The latter dimension is perceived as belonging to the adult realm.

In all the other cases, students appear not only able to express their core idea of time but they were also conscious that they were experiencing a special moment “that will be written in the history books” (Chiara) and that could be turned into something important for them. For example, Giorgia said:

“In this moment of suspension, the desire to be able to achieve something of personal success is even higher than before, so maybe it also gives that sense, that is, I self-influence my future.”

Alessandro said:

“I was struck by the fact that in a very short time the pandemic has completely changed the cards a little bit in general... [...] my perception of “long time” has been a bit modified... in perceiving how an event [...] can also quickly enter and rewrite a page of history…”.

The five complete appropriation profiles show that the conception of time outlined in the interview and reported in the previous section are very personal, authentic and genuine (marker A). The conception is deep in two senses: it is conscious (marker C) and non-accidental (marker D). Furthermore, it implies that a special role is ascribed to knowledge (marker B), as well as to positioning within a social network (marker E).

As we reported in the introduction, within the future shock induced by the society of acceleration, *alienation from time* is argued by Rosa to be strictly interrelated with *alienation from others* and *alienation from self*. The co-presence of the five markers in most of the students’ profiles shows that also the process of appropriation, the contrary of alienation, indicates that time appropriation is deeply connected with re-appropriation of the self (markers A, C and D) and of the relations with others (marker E). In our case, also the role of disciplinary knowledge (marker B) is stressed as a fundamental component of this complex process that relates time appropriation with identity formation (appropriation of self and of others).

In the following sections, we start showing the connection of markers A, C and D with the sense of the self, and then we move to unpack the marker related to the social dimension (marker E). Marker B is unpacked in the next section when we make a special focus on the role that science education plays or should play to equip the young to manage these deep social crises and transformations. In order to unpack the first set of markers, that is, the idiosyncratic idea (marker A), expressed by the students consciously (marker C) and in a non-accidental way (marker D), we apply three key concepts that we pointed out from the literature on sociology of time: the importance and the role of daily life rituals, the sense of agency on one’s own time and the sense of directionality. By re-reading students’ conceptions of time through these key concepts, the first finding we obtained is that all students’ conceptions are centred on personal daily life rituals. In fact, all the students express a revived need to stay in the present of their daily experiences and to become, first of all, agents of their personal daily lifetime.

The re-organizations of the daily life rituals are ways through which the students exert their agency to maintain an unchanged sense of self, that is, to maintain their intrinsic identity (diSessa 2018). Stefano, Giorgia and Gabriele were very keen on sustaining that the crisis did not really change their intrinsic identity: this emerged in their way of considering the lockdown as an opportunity to strengthen those routines or thoughts that they feel most “essential” (learning physics, training, observing “mankind”) and to legitimize them even further, for example, by awarding these rituals more time and more centrality in their daily life. Chiara, Alessandro and Laura perceived the change in a more significant way and had to change their rituals to preserve their inner sense of the self. Chiara had to invent new ways to control time and the new sense of oppression that she felt. Alessandro took the opportunity to widen his views and discover new perspectives from which society could be observed. Finally, Laura had to change profoundly the activities that generate her sense of time and, mainly, her sense of self.

Before the pandemic, sociological studies stressed that social acceleration had diminished the time of everyday life to trivial and repetitive rituals that slowly disappeared from the attention of our societies. From our students’ profiles, it appears that the conception of time during lockdown centralizes rediscovering daily time and its value. For all the students except Stefano, daily rituals are also the way to re-appropriate a deep sense of directionality that was lost in the era of future shock.

Giorgia is aware that, before the pandemic, we were in a moment of frenetic standstill, where change was somehow apparent. She feels the need to distinguish between “frenzy” and real development:

“Actually, I hope we understand the fact that this busy life didn't lead to so many developments in the end. [...] in this state, at least I have always felt it like this, everything was always the same, always constant over the years ... this situation and this block could give the opportunity for a change...”

For Giorgia, time has to be directional, and change has to be real: the past has to provide hints, and the present has to be exploited in order to become agent of one’s own future. Lockdown was an opportunity to rediscover and strengthen the directionality of time and make effective plans for the future.

Also for Laura, the student who most perceived the present shock, lockdown pushed her to search for a new sense of directionality. Her personal organization collapsed, and important events that she perceived as milestones for her future were cancelled. Thus, she had to reinvent her daily routines simply to re-appropriate a sense of directionality. Directionality became, for Laura, a process of cultivating her passions and exploring society to recognize how and where she can nurture her interests, now and in the future.

Alessandro has consciously recognized the frantic arrest as a change, but not as a problem: an opportunity to seek a new sense of directionality given by immersing in one’s own intellectual interests. Directionality for Alessandro, as for Laura, is offered by authentic intellectual thoughts or passions and by the sense they produce. For them both, directionality becomes lost in routines and experiences aimed solely at developing mere techniques. On the contrary, passions and sense-making give directionality, turning experiences into *Erfarunghen*. Laura uses her passions also to project herself into the future.

Gabriele is also looking for a non-routine routine. In his case, the search revolves around trying to “fill” time in the “right way”. Unlike Alessandro and Laura, Gabriele is not experiencing any sense of deep change, and he is filling the day with the same interests, activities and reflections. He lives in a sort of bubble that is in continuous interaction with the outside world and from which he observes society that, at this specific moment, has become aware of critical issues that have in fact always existed but which are now impossible to ignore. The pandemic confirmed to Gabriele his idea about the dynamics that propel society: people are capable of looking only at their own interests, at what is important in that moment, neglecting, most of the time, the true sense of things. However he recognizes that, for the world, the pandemic is a tragedy, and hence, he retains that “people” have to learn and find a sense of directionality: by looking at the past, people can extrapolate many teachings in order to achieve progress, better action in the future than in the present.

Chiara also perceives a sense of directionality in society but not in her private life. She manages the slowness and stillness of the present by immersing herself in bubbles of entertainment and imagination but perceives the directionality of the historic moment, where people are working hard to obtain a cure for the virus and, in general, to manage the crisis. As we already stressed above, Chiara feels as though her daily life is totally detached from the directionality of society, and for her personal life, the only thing she can do is escape within fictional scenarios. This is the dimension of present shock that Chiara is experiencing: a clear separation between the velocity, priorities and actions of the world outside and those of her daily life.

To sum up this first part of the analysis of students’ conceptions of time, aimed to unpack markers A, C, D through the lenses coming from sociology, we can stress two results: the idiosyncraticity appears not only in the choice of the rituals but also, and mainly, in the multiple forms assumed by the sense of directionality. Directionality and, more generally, the glance at the future are indeed the intrinsic aspect of time that mostly provides actions with a meaning. As we widely stressed in our framework on future shock, when directionality gets lost, time gets fragmented and identity becomes situational (Rosa 2013). The second result concerns the relation that emerged between daily life rituals, agency, and directionality. Such a strict relation indeed creates a strong demarcation line between the time of private life and the time that characterizes social phenomena. This is a result that challenged our expectations and one core aspect of the daily life rituals as they are described in the sociological literature. We expected to find, in daily life rituals, the manifestation of the nexus between the private and the public realm, between the personal and the societal dimensions. Instead, this extreme period of change revealed, at least in our group of students, the serious gap that exists between these dimensions.

This result allows us to unpack marker E, related to the social dimension. Also with respect to such a marker, students’ profiles of appropriation show interesting commonalities and diversities. This first commonality is that all the students perceive a significant misalignment between personal time and social time. This is very evident in Chiara and Gabriele. Chiara feels the oppression of the difference between the stillness of “suspended time” of her present and the speed of society that “moves super-fast”*.* Gabriele recognizes a different time structure of the personal and social time: the internal (personal) time is seen as made of “right non-routine routines”, whilst the external time, the “time of mankind”, is linear and deterministic and, he thinks, shows a sort of human inertia oriented toward avoiding any change.

In Stefano and Giorgia, the personal time creates a sort of “present bubble” that protects them from the external “storm.” Only Laura and Alessandro seem to perceive the need to maintain the nexus between the personal time of their experiences and social time. The realignment of their daily routines implies a dialogue with social changes and engagement with societal transformations. Alessandro said:

“I hope that [this present time] has made us understand the fragility of our whole system and of our way of life [...]. One of the reasons why we sometimes think that everything can't change, is stable, destined to last, looking at it from a single point of view, is because, maybe, there has never been a moment like this in which the whole system has broken down; and so I hope that we can all become aware [...] of the fact that it doesn't necessarily have to go on as we have done until now; in my opinion we won't come out [from this moment] with the answers but already changing the point of view of millions of people is a big step.”

Both Laura and Alessandro are very interested in social debate, and unlike Gabriele, they reveal a proactive mood toward the possibility of acting on society and taking some agency in societal transformations. This proactive mood was slightly more evident in Aldo’s interview (one of the three students that we interviewed first and that we did not consider for the profiles). Aldo was the only student who explicitly mentioned the concept of “responsible citizen” as the nexus between personal and social realms and the way individuals can take agency to contribute to a better world.

However, despite the references to society by Alessandro, Laura, Gabriele and Aldo, the students’ profiles highlight that the personal dimension is, as we might expect, the most relevant aspect in the period of isolation. Lockdown gave students the opportunity to rediscover and cultivate interests that had been neglected due to lack of time and to strengthen friendships and relations within the family. Furthermore, it also emerged that, for this group of talented students, ambition and trust in personal abilities were important ingredients in managing the crisis and exploiting the restored time. In our interviews, we saw only a few signals about the need to reflect on the role of individuals as responsible citizens and active agents in this global, fragile, and complex society. In accordance with the literature on future, dreams and hopes refer more to personal success than to a significant improvement of society as a whole. Even when society represents an important focus of attention, the perception that it can be changed and that each of us can be an agent of such changes seems, in general, faint. This is an important aspect where science education can insist and play a fundamental role.

To sum up, the *appropriation analysis* of the interviews confirms some intuitions we had about the benefits of reading the present as a moment where people feel deeply the need to re-appropriate their time. Here we took the markers we identified for describing the appropriation of a physical concept like temperature and extended their application to a more elusive concept like time. In the original approach the construct of appropriation allowed us to unpack how science learning could lead to a broader sense of learning and contribute to developing identity. Here, the application of our markers to time allowed us to recognize the elements characterizing time appropriation (the daily life rituals, agency and the sense of directionality) as foundational to rediscover or maintain one’s own identity and contribute to overcoming the sense of situationality (Rosa 2013). Moreover we recognized, in the diversity both of the rituals (e.g. the ritual of sense-making or rituals of skills development) and of the sense of directionality, the inner idiosyncratic aspects that regards the intrinsic identity (diSessa 2018). The multifaceted sense of directionality included both personal directionality given by a plan or by the search for sense and the collective directionality that society can find when huge events happen to upset the *status quo*. However the distinction of the two realms (personal and collective) and the sense of agency related mainly, if not only, to the first open up the main problem that we will address in the next section and that we can anticipate as follows: school and, in particular, science school has a very weak, if any, role to form any sense of social agency. Despite all the recommendations and the claims, our study confirms that school science and school education is mainly focused to prepare students as future (STEM, in our case) professionals than as social actors or responsible citizens.

### The Role of Science Education

The third axis we consider in comparing the profiles regards the role of science education. Such an axis allows us to unpack marker B and, mainly, to analyse the implications of the study both on school science and on the research in science education. The data we consider here come from those parts of the profiles designed to answer the following questions of the analytic grid: Does the student use disciplinary/school knowledge (particularly, scientific knowledge) to manage their time (grounded in the disciplines)? What contribution does the interview provide to build an argument about the roles of science education and disciplinary knowledge in equipping the students with knowledge and competences to manage fast-changing space and time structures?

Firstly, the six students’ profiles highlighted a polarized attitude toward the scientific knowledge acquired along their personal school path (*school science*). For three students out of six (Stefano, Chiara and Giorgia), school science represents a bubble in which they can feel at ease and that protects them from the toughness of reality. For example, for Stefano, who is a very good student, studying science and math and solving scientific problems are sources of intellectual pleasure. Also, scientific disciplines in their application are a source of escape: they help him to manage internal times to eliminate anxiety by distracting from the external problems. Stefano does not use science as a key to read and interpret reality and the contingency. It creates “isolated bubbles” that, like a game, provide pleasure, enjoyment, and social relationships. Also Chiara found in science a way to make sense of the present reality. In particular, the analysis of the epidemic curve from a mathematical perspective reassured and helped her to rationally manage the present. However, Chiara is aware that, in this moment, understanding the science behind the epidemic is important, but even more important is the need to personally manage the anxiety of the moment by escaping with her imagination. Even Giorgia finds a refuge in school science. Mathematics and physics are the subject matters that she prefers and their being, in her mind, a-historical and “certain” knowledge allows mathematics and physics school classes to restore a sense of “normality”. Classes continue just like before the pandemic, and the teacher does her best to “maintain normality” and do her classes as she did before lockdown. Giorgia thinks that this is possible because of the inner character of mathematics and physics as unchangeable, invariant, a-historical and certain disciplines. Thanks to these aspects, mathematics and physics school classes give Giorgia a sense of peacefulness, even in such a strong moment of crisis.

Despite the interest of those students toward science, their engagement with the discipline is not a key for interpreting the current debates. For the other three students (Gabriele, Alessandro, and Laura), school science represents a baseline knowledge to be refined with hints and reflections coming from other sources and/or to be enriched with extra-curricular activities. School science can set out, somehow, an initial mindset concerning the kind of methods and discourses that belong to science. For example, Gabriele holds scientific disciplines in high regard because they provide the basic knowledge that allows him to distinguish the person who holds the knowledge from the person who speaks to gain visibility or simply for the sake of speaking. School science allows him to decide who can be trusted or not, because it helps him to recognize when a discourse is well constructed and grounded.

Again, Alessandro recognizes the importance of scientific thought in this time of emergency and the role of scientific experts. However, he stressed that the study of scientific disciplines should provide not only knowledge—which may not be sufficient to enter into the merits of technical issues—but also the tools of scientific thought that allow him to move between the plurality of data and information and can act as a filter. Lastly, for Laura, physics and astrophysics are a deep passion on which she consciously builds her sense of time, by allowing her to project her imagination toward the future. Indeed, scientific methods and the science mindset are, for her, a way to rationally manage the present contingency and follow scientific controversies. However, this level of scientific awareness does not come from school science, which is completely removed from her mind, but from extra-curricular experiences.

We remind that the students selected for this study represent a sample of students chosen for bootstrapping the best information available about science education at school. All of them were highly motivated students, with a strong interest and passion for science, so much so that they had decided to attend a demanding extra-curricular course at the Department of Physics and Astronomy. Also because of the choice of sample for the study, the results have clearly revealed how much school science is missing an important educational opportunity in the pandemic era. Indeed, the patterns highlighted above, related to these two roles played by school science, can be seen as an expression of misalignment between the science taught at school and the science needed to deal with a “risk society” (Beck 2000).

For these students, school science is completely detached from society. Science they are taught remains isolated in a school context: at best, it influences their ways of thinking about their personal career or creates a space for enjoyment and self-accomplishment. Their ideas of science do not provide them with effective lenses to interpret social dynamics triggered by the pandemic crisis. Even in the cases of students who refer to society and pose problems with a mindset that could be interpreted as scientific, they do not recognize them explicitly as skills developed at school.

Another idea that emerges from students’ discourses is an epistemological belief that science heralds certain facts and indisputable truths that reassure and help them to make sense of the world. This could be ascribed to the almost-univocal focus of science curricula on Newtonian paradigm, where *determinism*, *linearity* and *prediction* are the keywords. The whole language of classical physics constructs an epistemological scaffolding to imagine the history and the future deterministically, as a linear progress toward an increasingly better world. Nevertheless, at the basis of the risk society, there is a very different paradigm, i.e. the complexity paradigm, where different temporal patterns and models of causal explanation are conceptualized. As we already mentioned when we presented the project I SEE in the background, the science of complex systems could be a very rich source of concepts like *scenarios*, *feedback*, *deterministic chaos* and *agent*, which could play crucial roles to open up new ways to conceive the scientific enterprise and its relationship with socially relevant themes like a pandemic. The concept of *uncertainty* could also be revalued in science education: not as something negative (lack-of-certainty) but as a concept that opens up a variety of possibilities for everyone’s imagination. Another important concept that complexity has at its core and that could be valued through education is the idea of *system*. Indeed, the recognition of the multiple levels that constitute a system allows the students to make explicit these levels and their relationships also about real-life contexts (e.g. individual, familiar, local, national, global levels). A system can be investigated not only for its levels but also for the dimensions it covers and impacts (e.g. scientific, economic, societal, personal, environmental, health). 

Developing rich discussions in the classrooms about these aspects could be important to foster competencies for analysis of complex issues. A STEM curriculum should include all these themes if we want to value science education as a way to prepare the young for this fast-changing society that appears more and more fragile and at risk.

## Discussion

In this paper, we borrowed an idea from sociology and used it to reflect on the role that science education can play to enable the young to navigate a fast-changing society. We started from the notion of future shock as characterization of the society of acceleration and observed that the pandemic has induced a different type of shock, which we define as “present shock”. The aspects that we took into account for characterizing this quick change are the fast disclosure of time structures that were invisible before the pandemic or diminished as trivial rituals. During a pandemic, the global time is accelerated by a natural phenomenon (the virus evolution), and we have become witnesses of the dynamics of social institutions (policy, health systems, educational systems, economics) trying to run as quickly as possible to slow down the speed of the epidemic. At the same time, during lockdown, our individual time was suspended in a bubble of present that had to be redefined in its inner rituals.

On the basis of this idea, we carried out an empirical qualitative study to investigate how teenagers with a strong affinity for science were experiencing this moment of change and what could we learn from them, both to better define the idea of present shock and to reflect on the role of science education. Our study focuses on six interviews and has a merely exploratory and piloting character: the type and number of the students makes the sample very particular but also, in our opinion, particularly rich for paving the way for further, wider, and more specific, rounds of investigation.

We carried out a four-phase analysis of the six interviews. First we searched for the *sensitizing concept* by navigating the whole corpus of data, and we were able to identify that perception of future was not the way to encode the time structures that the students were enacting to manage the current situation. This phase led us to recognize, instead, the potential of our appropriation markers, and we designed our analytic grid accordingly (Phase 2). The dichotomy between *alienation from time* and *time re-appropriation* became the crucial aspect for highlighting the difference between future shock (characteristic of the society of acceleration) and present shock (characteristic of the pandemic era).

The application of the appropriation markers led us to organize the data into six comparable profiles (Phase 3), which we analyzed and discussed along three different axes: (i) students’ conceptions of time, (ii) appropriation and the nexus between time conception and identity and (iii) the role attached by the students to science and its learning (Phase 4).

Each comparison led us to recognize inner but important aspects of students’ discourse.

First of all, we saw that the macro-distinction between space and time as *containers* and space and time as *relations*, which characterizes the debate on space and time in physics and philosophy, is always effective in capturing the main idea of time (and space). However, we also saw that we needed to refine the lens to restore, faithfully, the richness of students’ discourse. We then used criteria taken from the construct of appropriation elaborated within science education to capture authenticity in disciplinary learning.

The application of our markers allowed us to argue that appropriation is in fact an effective counterpart of alienation. As *alienation from time* is strictly interrelated with *alienation from others* and *alienation from self*, also the process of time re-appropriation during the era of pandemic is strictly related to the appropriation of self (markers A, C and D) and of the relations with others (marker E). In order to unpack markers A, C and D, we used criteria taken from the sociology of time: the role of daily life rituals, agency on one’s own time and directionality. These criteria allowed us to connect the conception of time with the notions of intrinsic (diSessa 2018) and situational identity (Rosa 2013). More specifically, we were able to identify that students’ conceptions of time were built on daily lifetime rituals that the students enacted to maintain an unchanged sense of self (their intrinsic identity). In some cases, the maintenance of intrinsic identity implied simply allocating more time and priority to routines, activities, or thoughts regarding authentic interests (like reading, training, or thinking). In other cases, it required a deep and, somehow, “dramatic” rearrangement of daily rituals. Independently of the individual differences, our analysis shows that, within moments of deep change, the time structures that contribute to forming identity become particularly evident. During a period of lockdown, each of us, more and more explicitly, dealt with the need to realign self-perception as agents or receptors of change and sense-making producers and to adjust the ways we relate with others. This complex process requires the recruitment and application of a great deal of knowledge (diSessa 2018), but also, we saw specific time structures. Identity process is indeed regulated by rituals and routines that shape our “reflective project”, made up of selective processes, exploration of options and decision-making processes. Moreover, the application of our markers to time allowed us to recognize two further elements in the time rituals that are related to identity and, specifically, to a deep sense of identity that is *not* situational: the sense of agency and directionality. The discovery of a multifaceted sense of directionality makes this aspect deeply related to intrinsic identity and shows where the young tend to act to re-appropriate their future and the sense of their learning and their actions. This aspect is mainly the most relevant finding of the study that pointed out a serious detachment between a sense of personal directionality and of social directionality.

Students’ discourse revealed a special dialogue with oneself and “others” (marker E). Specifically, we discovered that the “others” in students’ discourse are friends, parents, teachers, and influencers. There is not that chorality that one could recognize as a sense of “collectiveness”, of “society”. This revealed a break and deep detachment in the personal and the social dimension: the rules and paces that characterize daily life personal rituals on which they feel to be agents appear completely different and detached from what the students perceive to be the dynamics of society. Some of them use science to build and stay in their own space-time bubble, protected from society. The social directionality, even when it is perceived or recognized, is far from their agency.

As for personal directionality, one aspect emerged that we found deeply problematic: students manifested in the interviews a strong, urgent and authentic need to immerse themselves in deep personal experiences, but in several cases, this need was completely detached from what the school offers or requires them to do. This remark moves us on to the final comments. The school emerged from the interviews as a source of normative and soothing rituals that, even in a pandemic era, can offer certainties and a sense of normality. This is particularly true for the classes of mathematics and physics, i.e. the favourite subject matters of these students. They are seen either as sources of a-historical, unchangeable knowledge whose teaching cannot be affected even by such a deep crisis or as a solid baseline on which personal or social interests can be nurtured by extra-curricular activities. From this picture and from our analysis, we see that science and STEM teaching could play definitively at least three more relevant roles. Firstly, time and space are scientific themes that scholars from all other fields (sociology, philosophy, literature, policy, etc.) use to interpret complex phenomena occurring in our global and fragile society. It is a missed opportunity that science and STEM curricula do not dedicate special attention to them, in terms of language, structures, and epistemologies elaborated to conceptualize and manage them. Time and space are intrinsic interdisciplinary themes that, if properly addressed, can turn knowledge into fundamental competencies for the twenty-first century. In our previous projects, the debate on space and time was at the basis of the design of our materials on relativity, and in the project I SEE, we designed modules to point out the causal structures that science elaborated for managing uncertainty and the future in terms of forecasting or foresight. As we mentioned, the science of complex systems is an impressive source of fundamental concepts for consciously navigating the risk society. We are now working, within the project IDENTITIES, to refine these modules and exploit their interdisciplinary character (www.identitiesproject.eu).

Secondly, in this fast-changing society, it is more and more urgent that science education prioritizes research studies aimed at evaluating its role in developing students’ identities. This study shows that this also implicates revision of the curricula and teaching materials so as to boost specific rituals: training routines aimed at developing technical skills but also, and mainly, rituals explicitly aimed at breaking down routines and fostering immersion in personal interests and thoughts. This operationally means designing materials and enacting teaching strategies that enable students to play with their own ideas and make them feel that their personal search for sense is legitimate and welcome also within the classroom culture. In our study on appropriation, we argue how materials built on multidimensionality, multi-perspectiveness and longitudinality can support classroom dynamics that foster appropriation (Levrini et al. 2019a, 2020a ). Now, we have unveiled new time properties that should hold in order to foster identity formation. These properties link school and extra-curricular (home) activities, regarding the role of daily rituals and the centrality of a sense of directionality.

Thirdly, school science has to thoroughly redefine its institutional role within society. Not only the knowledge but also the rituals that science teaching tends to boost seem to produce the effect of creating “bubbles”. In a society characterized by a communication that enhances polarization and echo chambers, the school system plays a crucial role and can make the difference. School must open up to society and enact processes that, whilst contributing to forming students as active and responsible citizens, also transform itself as an institution. This is the main direction that this study will follow in our next research studies within two Horizon 2020 projects: the SEAS project (https://www.seas.uio.no) and the recently approved FEDORA project. We are indeed in an extraordinary period and must not miss the opportunity for deep social transformation toward a more human, rational, equal, and sustainable society where there is space and time to take care of ourselves, others, and the planet.

## Appendix 1. Annex - Interview Protocol

We are going through a very particular moment that is changing, among many other things, our daily lives, the ways to deal with our relationships and our perception of time.

### Section 1. Questions about the management of the present

1. How do you concretely organize your day? What are the new, most difficult and most surprising aspects?
2. Is there anything you do with particular pleasure in this period? If so, what is it?
3. Is there anything in particular that scares or disorients you? If so, what is it?
4. Among the subjects you study at school, is there anything you pleasantly study (e.g. history, philosophy, science, literature, art)?
5. Outside school, are there any activities you do with particular pleasure (e.g. reading, playing or listening to music, following debates on political/economic/social issues)? Are there specific platforms/channels where you carry out these activities (e.g. social networks, television, associations)?
6. Are you following debates that help you to interpret this historical moment? If so, which debates are you following, on which topics (e.g. health, political, philosophical, artistic, economic, psychological, social, artistic issues)? Which platforms/channels do you use to follow them (e.g. social networks, television, associations)?
7. Are there any experts you refer to? If so, what type of expert (e.g. teachers, scientists, medical/health personnel, politicians, journalists, writers, philosophers, communicators, friends, influencers)? Are there any expert persons in particular? If so, who?
8. Is there anything you have learned in this particular period that you consider important for your personal, cultural and professional growth? If so, what?
9. More specifically, have you learned anything important about how science proceeds? If so, what? Have you learned anything about the relation between science, society and politics? If so, what?
10. Has your relationship of trust toward experts changed? If so, how?
11. On which dimensions of the problem do you tend to reflect more often (e.g. personal, emotional, scientific, political, ethical)?
12. Do you think that your knowledge of science, mathematics and physics can play a role for understanding and managing this situation? If so, which role? If not, why?

### Section 2. Questions about the perception of time (present, past and future)

13. Do you feel more like a present, past or future person? That is, to manage a moment of change, do you find greater stimulus/trust/refuge in the study of history and in drawing lessons from the past, in the management of present contingency or in imagining/dreaming future scenarios? Can you explain in what sense?
14 Has this crisis changed your perception of the “small” time of your daily life? If so, how? Do you feel empty times, times of boredom, of waiting in your daily life? Do the days seem longer?
15 Do you feel acceleration times or pressures from the outside that influence your daily life?
16 Has this crisis changed your perception of the “big time” of history? If so, how?
17 Do you think the world will emerge better or worse from this crisis? How?
18 Will the future of society benefit from this or should we expect a worsening compared with the future that was expected before the crisis?
19 Has this crisis changed your perception of the “mean” time of your education (e.g. choice of university, choice of professional field, choice of career)?
20 We give you three different time horizons: the end of the lockdown, the end of school and the end of university. How do you imagine yourself at these times? In which scenarios, or in which global social/political/cultural contexts, do you imagine yourself?
21 Do you think this moment of emergency will affect your future? What future? How?
22 Do you think this moment of emergency will affect society as a whole? If so, in which aspects?
23 How would you define your mood in front of this great change?
